# The Role of the Diaphragm in Postural Stability and Visceral Function in Parkinson’s Disease

**DOI:** 10.3389/fnagi.2021.785020

**Published:** 2021-12-23

**Authors:** Xin Yu, Hong-ying Jiang, Chen-xi Zhang, Zhao-hui Jin, Lei Gao, Rui-dan Wang, Jin-ping Fang, Yuan Su, Jia-ning Xi, Bo-yan Fang

**Affiliations:** ^1^Beijing Rehabilitation Medical College, Beijing Rehabilitation Hospital, Capital Medical University, Beijing, China; ^2^Department of Respiratory Rehabilitation Center, Beijing Rehabilitation Hospital, Capital Medical University, Beijing, China; ^3^Parkinson Medical Center, Beijing Rehabilitation Hospital, Capital Medical University, Beijing, China

**Keywords:** Parkinson’s disease, diaphragm, postural stability, visceral function, motor subtypes

## Abstract

**Background:** In normal subjects, the diaphragm plays a key functional role in postural stability, articulation, respiration, defecation, and urination.

**Objectives:** The aim of this study was to investigate the role of the diaphragm in postural stability and visceral function in patients with Parkinson’s disease (PD) and to compare the diaphragm function by gender, Hoehn and Yahr (H&Y) staging, and motor subtypes.

**Methods:** In total, 79 patients were enrolled in this cross-sectional study. The severity of the disease was assessed by the Movement Disorder Society-Unified Parkinson’s Disease Rating Scale III and by H&Y staging. Postural stability was quantitatively recorded, and respiratory function was evaluated by spirometry. Several scales were used to evaluate visceral function in patients with PD. In addition, diaphragm ultrasound was used to measure the excursion, contraction velocity, and thickness of the diaphragm during quiet breathing, deep breathing, and the sniff test. Significant features were selected by the least absolute shrinkage and selection operator (LASSO) regression and fitted in the multivariate linear regression and Pearson’s correlation analysis.

**Results:** Diaphragm thickness and excursion during quiet breathing were significantly different between men and women and between H&Y stage 1–2 and stage 2.5–3, whereas the diaphragm function was not influenced by motor subtypes. It was shown that the diaphragmatic function was significantly correlated with postural stability, voice function, respiratory function, constipation, and urological function to varying degrees in patients with PD.

**Conclusion:** The diaphragmatic function is associated with dysfunction in PD although it remains unclear as to whether the observed changes in the diaphragm are primary or secondary.

## Introduction

Parkinson’s disease (PD) is a common neurodegenerative disease that is associated with major motor symptoms, including resting tremors, rigidity, bradykinesia, and postural instability. Patients with postural instability are more likely to fall, leading to reduced mobility and life expectancy (Fasano et al., 2017). PD involves the accumulation of α-synuclein in multiple visceral systems which play essential roles in the non-motor symptoms (Pfeiffer, 2020), including dysarthria (Schalling et al., 2017), delayed gastric emptying and constipation (Fasano et al., 2015), urinary urgency, and nocturia (Jia et al., 2020). If not treated appropriately, the combination of motor and non-motor symptoms can significantly reduce the quality of life (QoL; Schapira et al., 2017). In addition, researchers have discovered abnormalities in the respiratory center during the early stages of PD that appear to arise from α-synuclein accumulation in the dorsolateral pons and ventrolateral medulla (Zhang et al., 2019).

The diaphragm is a muscle pump that plays a key role in generating negative intrathoracic pressures for ventilation and positive intra-abdominal pressures for expulsive behaviors and venous return. The non-ventilation function of diaphragm has been discussed the diaphragm generates no more than 30% of the maximal transdiaphragmatic pressure to maintain ventilation requirements during exercise while to perform expulsive maneuvers such as defecation and parturition, diaphragm generates almost maximal transdiaphragmatic pressures, which suggest the reserved capacity of the diaphragm for certain functions (Fogarty and Sieck, 2019). Specifically, although limits of stability (LOS) were significantly reduced in the elderly compared to the healthy young group, the diaphragm was thicker in the elderly, and the muscle thickness also had a positive correlation with LOS. The abovementioned findings from previous research may indicate the compensatory role of the diaphragm in postural stabilization (Özkal et al., 2019). Regarding the role of the diaphragm in phonation, the posterior and middle diaphragm is the main part to elevate and act as one functional unit to keep constant subglottic pressure during phonation (Traser et al., 2017). In terms of visceral function, the diaphragm, pelvic floor muscles, and abdominal muscles synergistically operate to maintain intra-abdominal pressure and accommodate to the changes in the abdominal cavity, for example, with increasing gas load in the colon, the diaphragm relaxes whereas the anterior abdominal wall increased the tone to enlarge intra-abdominal volume in healthy volunteers (Villoria et al., 2011). Besides, diaphragmatic breathing training in combination with strength training has also been demonstrated to improve overactive bladder, which may be associated with the activation of deeper muscle and the reduction of detrusor pressure (Hagovska et al., 2020).

Parkinson’s disease is also a heterogeneous disease that could be classified into tremor dominant (TD) subtypes/indeterminate subtypes/postural instability and gait disorders (PIGD) subtypes according to the Movement Disorder Society-Unified Parkinson’s Disease Rating Scale (MDS-UPDRS; Stebbins et al., 2013). Researches have demonstrated that such classification is more suitable in that it comprehensively reveals significant differences between different motor subtypes related to the activity of daily living, motor symptom severity, and cardiovascular and gastrointestinal symptoms (Ren et al., 2020). The progression of PD is often described using the Hoehn and Yahr (H&Y) staging, which weighted the bilateral motor symptoms involvement and balance control as the principal mark of disease severity (Hoehn and Yahr, 1967). The modified version of the H&Y scale introduced 0.5 increments to evaluate disease progression (Goetz et al., 2004).

Diaphragm function is characterized by diaphragm thickness during tidal breathing, diaphragm excursion during three maneuvers (i.e., quiet breathing, sniff test, and deep breathing), and diaphragm contraction velocity during quiet breathing and sniff test. The criteria of normal diaphragm function are based on the research from Sarwal et al. (2013) and Tuinman et al. (2020), in which male and female subjects always exhibit different standards. The objective of this study was to (1) compare the diaphragm function in patients with PD by gender, motor subtypes, and disease staging and (2) investigate the correlation between the diaphragm and postural stability, articulation, respiration, defecation, and urination in patients with PD.

## Materials and Methods

### Participants

This cross-sectional study is part of an ongoing project (active and passive biofeedback and neuromodulation collaborative therapy system evaluation and clinical validation, ChiCTR1900020771, registered on January 19, 2019). In brief, we screened 436 patients between February 2019 and December 2020 from outpatient and the internet. Of note, 81 eligible patients were included in the project of the Parkinson’s Medical Center, Beijing Rehabilitation Hospital, Capital Medical University, and two patients were dropped out (Supplementary Figure 1). We recruited patients between 30 and 75 years of age and in accordance with the clinical diagnostic criteria from the MDS (Postuma et al., 2015), including clinically established PD and probable PD with H&Y stage ≤3. The study was approved by the Ethics Committee of Beijing Rehabilitation Hospital of Capital Medical University (2018bkky022), and each patient signed an informed consent form.

We excluded patients with atypical Parkinsonism and those who had comorbidities, including cardiac diseases, respiratory diseases, and neurological diseases. A range of baseline information was collected including age, course of the disease, gender, H&Y stage, and 39-item Parkinson’s Disease Questionnaire (PDQ-39). Motor subtypes were determined according to the method proposed by Stebbins et al. (2013), patients were characterized as TD subtypes if TD/PIGD ratio ≥1.15, if TD/PIGD ratio ≤0.9 then patients were considered as PIGD subtypes, and patients with a ratio between 0.9 and 1.15 were indeterminate.

### Evaluation of Motor Function and Postural Stability

Motor function was evaluated by the MDS-UPDRS III with the same qualified physiotherapist. Postural stability was evaluated by the Stability and Balance Learning Environment (STABLE) system (Motek, Amsterdam/Culemborg, Netherlands) without visual feedback. The mean velocity of the center of pressure (COP) was determined in the following three conditions during the “ON” state: standing on both feet with eyes open, standing on both feet with eyes closed, and tandem standing. During the evaluation, the patients were instructed to look ahead and to avoid using handrails. These evaluation methods provide us with information on static balance and postural stability in the patient’s standing posture.

### Evaluation of Visceral Function

Evaluation of respiratory function: All patients with PD were seated and wore nose clips to perform pulmonary function tests delivered by a skilled respiratory physician. Forced vital capacity (FVC) maneuver and vital capacity (VC) maneuver were conducted in accordance with the guidelines from the American Thoracic Society and the European Respiratory Society (Graham et al., 2019). VC, FVC, forced expiratory volume during the first second (FEV_1_), and FEV_1_/FVC were recorded.

Evaluation of voice function: The Voice Handicap Index-10 (VHI-10) is commonly used to evaluate voice function in patients with PD, which consists of five physical, three functional, and two emotional items (Sunwoo et al., 2014). Participants were scored based on the frequency of symptoms ranging from 0 (never happened) to 4 (always happens); therefore, a higher score indicated that the perceived symptoms were more severe. Due to the cultural differences between eastern and western societies, the physiological aspect accounts for a greater proportion in the simplified Chinese version (Li et al., 2012).

Evaluation of intestinal function: The Bowel Function Index (BFI) is a physician-scored assessment incorporating defecation difficulty, the perception of incomplete evacuation, and the overall judgment of constipation over the last week (Ducrotté and Caussé, 2012). Patients arbitrarily chose any number from 0 to 100 according to the severity of constipation for each question. The BFI is the mean score of three questions. The Patient Assessment of Constipation Quality of Life (PAC-QoL) is a 5-point Likert-type scale that consists of four subscales, including worries and concerns, physical suffering, mental suffering, and dissatisfaction (Marquis et al., 2005). A higher score in BFI or PAC-QoL indicated that the subjective judgment of constipation or related burden was more severe.

Evaluation of urological function: The International Consultation on Incontinence Questionnaire for Overactive Bladder (ICIQ-OAB) was used to evaluate the severity of lower urinary tract symptoms in patients with PD, including domains related to frequency, urgency, nocturia, and urinary incontinence (McDonald et al., 2020). The International Prostate Symptom Score (IPSS) was also used to evaluate the filling (three items) and voiding (four items) phases in patients with PD (Brusa et al., 2020). For both of these questionnaires, a higher score implied more severe symptoms in the lower urinary tract.

### Ultrasound of the Diaphragm

**FIGURE 1:**
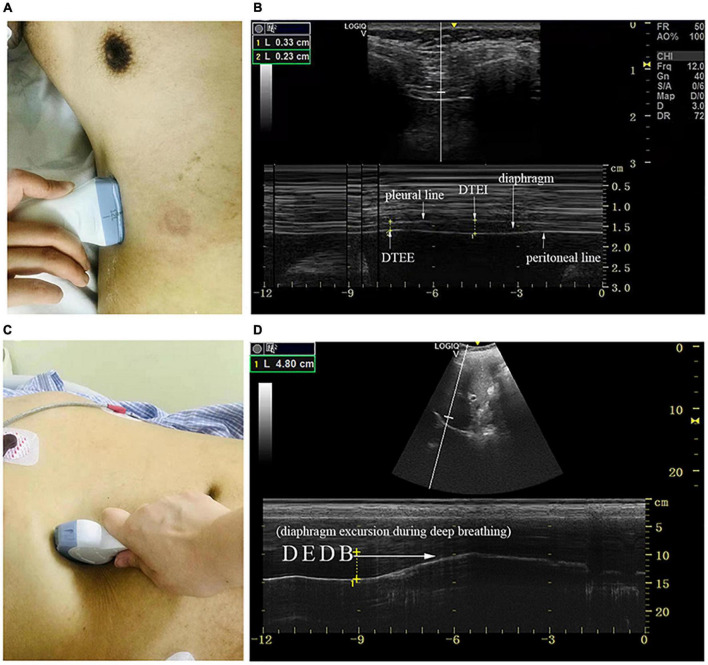
The measurement of diaphragmatic thickness and excursion. **(A)** A 10–15 MHz probe was placed at the zone of apposition. **(B)** The non-echogenic layer between the yellow markers indicates the thickness of the diaphragm at the end of expiration and inspiration. **(C)** A 2–5 MHz curved-array probe was placed under the costal margin. **(D)** The bright line indicates diaphragmatic excursion during deep breathing. DTEE, diaphragm thickness at end-expiratory; DTEI, diaphragm thickness at end-inspiration; DE_DB_, diaphragmatic excursion during deep breathing.


Diaphragm ultrasound (GE, LOGIQ e, Germany) was performed by a skilled respiratory physician with ultrasound experience to measure bilateral excursion and contraction velocity as well as the thickness of the diaphragm during different maneuvers. Patients remained in the supine position during the examination. The thickness of the diaphragm was determined with a 10–15 MHz linear transducer placed perpendicular to the skin over the zone of apposition to the rib cage, between the midaxillary and antero-axillary line on both sides (Figure 1A; Vivier et al., 2012). In the zone of the apposition, the abdominal organs were observed to reach the bottom of the rib cage where the diaphragm was bounded by two echogenic layers (i.e., peritoneum and the diaphragmatic pleurae) (Figure 1B). The thickness of the diaphragm was defined as the distance between the peritoneum and the diaphragmatic pleurae. We recorded diaphragm thickness at end-expiration (DTEE) and end-inspiration (DTEI) until at least three breathing cycles had been captured. The diaphragm thickening fraction (DTF) was calculated by the following equation.

(1) $$\text{DTF}=\frac{\left(\text{DTEI}-\text{DTEE}\right)}{\text{DTEE}}\times 100\%$$

Diaphragm excursion was determined with a 2–5 MHz curved-array probe under the costal margin in the midclavicular line and perpendicular to the diaphragmatic dome on both sides of the body where the liver and spleen served as the acoustic window (Figure 1C). M mode was chosen to demonstrate the diaphragm excursion during quiet breathing (DE_QB_), the sniff test (DE_Sniff_), and deep breathing (DE_DB_) (Figure 1D). The excursion was defined as the maximal perpendicular distance of diaphragm movement. We also determined the diaphragmatic contraction velocity during quiet breathing (DV_QB_) and the sniff test (DV_sniff_).

### Statistical Analysis

Numerical variables were described as means ± SD or as medians and interquartile ranges according to the results of Kolmogorov–Smirnov or Shapiro–Wilk normality tests. Mean imputation was applied for missing values. Differences in the diaphragmatic parameters between gender and motor subtypes were assessed by the Mann–Whitney *U* test and the Kruskal–Wallis test; differences between H&Y stage 1–2 and stage 2.5–3 were also compared using the independent *t*-test or the Mann–Whitney *U* test; *p* < 0.05 was regarded as being statistically significant; and 95% CI was also analyzed. Statistical analysis was carried out with SPSS version 25.0 (IBM, Armonk, NY, United States). To overcome the data overfitting and select the main diaphragmatic variables, the least absolute shrinkage and selection operator (LASSO) regression was performed using the “glmnet” R package (Friedman et al., 2010). The regression model was determined when the penalty parameter lambda (λ) was minimum or plus one standard error. In one model related to postural stability (tandem standing), significant features were first fitted into the principal component analysis to exclude collinearity. Pearson’s correlation analysis and multivariate linear regression were also used to determine the correlation between the diaphragmatic parameters and the dependent variables (i.e., postural stability, VHI-10, spirometry test, BFI, PAC-QoL, ICIQ-OAB, and IPSS).

## Results

A total of 81 patients with PD were enrolled; two patients were dropped out. Demographic baseline information, such as disease duration, H&Y stages, and PDQ-39 score, was summarized in Table 1. The means of MDS-UPDRS III scores in the OFF and ON states were 38.87 and 30.57, respectively. The similar scores could be related to the fact that mild-to-moderate patients with PD may not manifest with motor fluctuations. Motor function and visceral function were also presented in Table 1.

**TABLE 1:** Clinical features and diaphragm function in patients with Parkinson’s disease.

Characteristic	Overall Cohort (*n* = 79)	Male	Female	*P* value	Abnormal value
**Demographic characteristics**					
Age (year)	61.5 (9)	64 (13)	61 (8)	0.2	/
Disease duration (months)	78 (60)	6 (5.42)	7 (4.3)	0.5	/
Gender (male), n (%)	35 (44.3%)	35 (44.3%)	44 (55.7%)		/
Hoehn & Yahr stage, n (%)				0.6	/
Stage 1–1.5	6 (7.6%)	4	2		/
Stage 2	39 (49.4%)	18	21		/
Stage 2.5	16 (20.3%)	7	9		/
Stage 3	18 (22.8%)	6	12		/
PDQ-39 (score)	23.07 (13.46)	21.15 (11.3)	25.96 (12.83)	0.02*	/
MDS-UPDRS III-ON (score)	30.57 ± 12.48	31.17 ± 13	30.67 ± 11	0.9	/
MDS-UPDRS III-OFF (score)	38.87 ± 14.17	37.68 ± 12.2	38.12 ± 14.6	0.9	/
**Postural stability (COP)**					
Eyes open (cm/s)	2.78 (0.98)	2.5 (1.1)	2.8 (0.9)	0.2	/
Eyes closed (cm/s)	2.83 (0.86)	2.7 (1.0)	2.9 (0.7)	0.14	/
Tandem standing (cm/s)	4.16 (1.90)	5.3 (2.3)	3.8 (1.1)	< 0.001*	/
**Voice function**					
VHI-10 (score)	8 (7)	9 (8)	7 (7)	0.14	>7.5
**Respiratory function**					
VC (% of predicted)	100.48 ± 18.76	93.67 ± 14.56	105.89 ± 27.8	0.02*	<80
FVC (% of predicted)	95.47 ± 15.40	92.23 ± 14.22	98.03 ± 22.5	0.1	<80
FEV_1_ (% of predicted)	91.10 ± 14.82	90.36 ± 13.67	91.61 ± 15.81	0.7	<80
FEV_1_/FVC (%)	78.12 ± 4.94	75.8 (6.5)	79.7 (5.2)	0.2	<70
**Intestinal function**					
BFI (score)	46.67 (53.33)	56.7 (53.3)	40 (48.75)	0.2	>29
PAC-QoL (score)	48 (20.05)	54 (37)	47 (31)	0.5	/
**Urological function**					
ICIQ-OAB (score)	4 (3)	4.8 (4)	4 (2)	0.4	Mild: 0–7; Moderate: 8–19; Severe: >20
IPSS (score)	8 (13)	8 (13)	/	/	
**Diaphragm function**					
DTEE (cm)	**0.21 (0.09)**	**0.23 (0.06)**	**0.20 (0.08)**	0.005*	Men: <0.17; Women: <0.13
DTEI (cm)	**0.34 (0.14)**	**0.4 (0.15)**	**0.30 (0.13)**	< 0.001*	
DTF (%)	62 (43)	68 (56)	57.5 (42)	0.26	<20
DE_QB_ (R) (cm)	**1.2 (0.65)**	**1.45 (0.6)**	**1.13 (0.54)**	0.028*	Men: <1; Women: <0.9
DE_Sniff_ (R) (cm)	4.35 ± 1.47	4.63 ± 1.51	4.12 ± 1.42	0.13	Men: <1.8; Women: <1.6
DE_DB_ (R) (cm)	4.89 ± 1.32	5.20 ± 1.43	4.64 ± 1.19	0.06	Men: <4.7; Women: <3.7
DV_QB_ (R) (cm/s)	1.07 (0.32)	1.09 (0.32)	1.02 (0.30)	0.13	/
DV_Sniff_ (R) (cm/s)	3.77 (1.8)	3.98 (2.34)	3.66 (1.39)	0.40	/
DE_QB_ (L) (cm)	1.24 (0.53)	1.33 (0.74)	1.21 (0.48)	0.24	/
DE_Sniff_ (L) (cm)	3.96 ± 1.3	4.02 ± 1.39	3.92 ± 1.24	0.74	/
DE_DB_ (L) (cm)	4.19 ± 1.23	4.16 ± 1.34	4.22 ± 1.15	0.82	/
DV_QB_ (L) (cm/s)	1.05 (0.41)	1.05 (0.49)	1.05 (0.37)	0.64	/
DV_Sniff_ (L) (cm/s)	3.78 (1.62)	3.57 (1.97)	3.78 (1.26)	0.45	/

Data are expressed as means ± SD if normally distributed; otherwise, data are shown as medians with interquartile ranges (IQRs). *p < 0.05 (two-tailed) defined as being statistically significant and was shown in bold. n, number; PDQ-39, 39-item Parkinson’s Disease Questionnaire; MDS-UPDRS, Movement Disorder Society-Unified Parkinson’s Disease Rating Scale; COP, center of pressure; VHI-10, Voice Handicap Index-10; VC, vital capacity; FVC, forced vital capacity; FEV_1_, forced expiratory volume during the first second; BFI, Bowel Function Index; PAC-QoL, Patient Assessment of Constipation Quality of Life; ICIQ-OAB, International Consultation on Incontinence Questionnaire for Overactive Bladder; IPSS, International Prostate Symptom Score; R, right; L, left; DTEE, diaphragm thickness at end-expiratory; DTEI; diaphragm thickness at end-inspiration; DTF, diaphragm thickening fraction; DE_QB_, diaphragmatic excursion during quiet breathing; DE_Sniff_, diaphragmatic excursion during sniff test; DE_DB_, diaphragmatic excursion during deep breathing; DV_QB_, diaphragm velocity during quiet breathing; DV_Sniff_, diaphragm velocity during sniff test.

**FIGURE 2:**
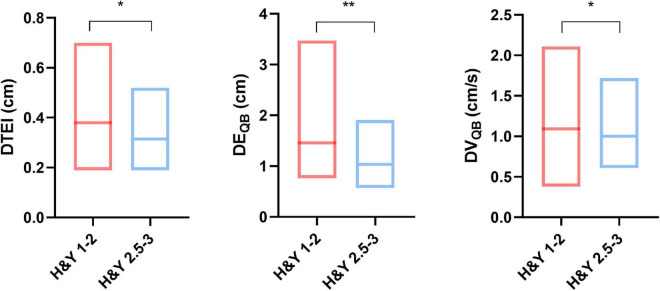
Comparison of diaphragm function between different Hoehn and Yahr (H&Y) stages. *p < 0.05; **p < 0.01. DTEI, diaphragm thickness at end-inspiration; DE_QB_, diaphragmatic excursion during quiet breathing; DV_QB_, diaphragm velocity during quiet breathing.

Diaphragmatic parameters were divided into three categories, namely, thickness, excursion, and velocity. We not only presented the data from the overall cohort but also compared the data by gender (Table 1), H&Y stage (Figure 2), and motor subtypes (Supplementary Table 1). Compared with women, both diaphragmatic thickness and excursion during quiet breathing were significantly higher in men. The diaphragm function of patients with PD was beyond the pathological values. Patients in H&Y stage 1–2 presented significantly higher diaphragm thickness at the end of inspiration (0.38 ± 0.12 vs. 0.32 ± 0.09, *p* = 0.025), higher diaphragm excursion [1.46 (0.52) vs. 1.03 (0.57), *p* = 0.007], and higher contraction velocity during quiet breathing [1.09 (0.33) vs. 1 (0.34), *p* = 0.03] (Figure 2). In contrast, we did not find any significant differences in diaphragm function between motor subtypes. In addition, the right-to-left ratio during quiet breathing was within the normal but was higher in several patients during deep breathing (Supplementary Table 2).

**FIGURE 3:**
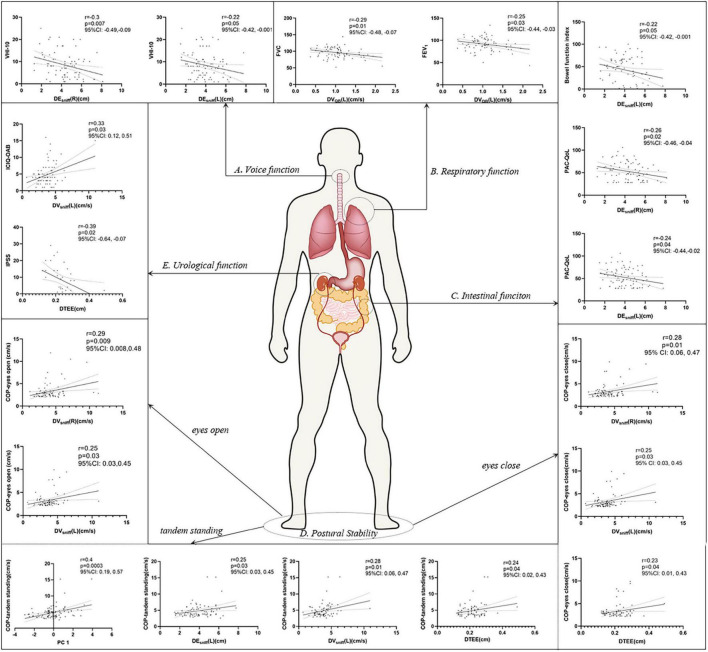
Pearson’s correlation analysis between the diaphragm and voice **(A)**, respiratory **(B)**, intestinal **(C)**, postural stability **(D)**, and urological function **(E)**. *p* < 0.05 was considered to be statistically significant. VHI-10, voice handicap index-10; PC, principal component; FVC, forced vital capacity; FEV_1_, forced expiratory volume during the first second; BFI, Bowel Function Index; PAC-QoL, Patient Assessment of Constipation Quality of Life; ICIQ-OAB, International Consultation on Incontinence Questionnaire for Overactive Bladder; IPSS, International Prostate Symptom Score; COP, center of pressure.

In a further analysis, we selected several principal features through the LASSO regression (Supplementary Figure 2 and Supplementary Table 3) and entered those features in the multivariate linear regression analysis (Table 2). Diaphragm function was a significant predictor for static balance, voice, and respiratory function, while diaphragm together with age was determined as the significant predictors for intestinal and urological function. Pearson’s correlation analysis (Figure 3 and Supplementary Table 4) revealed that the contraction velocity of the diaphragm was positively correlated with postural stability during eyes open (r/p 0.29/0.009) and eyes closed (r/p 0.28/0.01). The diaphragmatic principal component extracted from excursion and contraction velocity was also positively correlated with postural stability during tandem standing (r/p 0.4/0.0003). In contrast, VHI-10 showed a negative correlation with a diaphragmatic excursion (r/p −0.3/0.007; −0.22/0.05). Besides, we also found significant correlation between diaphragm and respiratory function [i.e., FVC (r/p: −0.29/0.01) and FEV_1_ (r/p: −0.25/0.03)], BFI (r/p −0.22/0.05), and PAC-QoL (r/p −0.26/0.02; −0.24/0.04). Furthermore, diaphragm contraction velocity was also positively correlated with the ICIQ-OAB scores (r/p 0.33/0.003) whereas thickness was negatively correlated with the IPSS scores (r/p −0.39/0.02).

**TABLE 2:** Main diaphragmatic parameters associated with postural stability and visceral function selected by the least absolute shrinkage and selection operator (LASSO) regression and the principal component analysis and fitted in the multivariate linear regression.

	Adjusted *R*^2^	Standard β coefficient	*p* value
**Postural stability (COP)**			
**Eyes open**	0.08		
DV_sniff_ (R)		0.29	0.009*
**Eyes close**	0.06		
DV_sniff_ (R)		0.28	0.01*
**Tandem standing**	0.15		
PC 1		0.4	< 0.001*
**Voice function**	0.08		
DE_sniff_ (R)		–0.3	0.007*
**Respiratory function**			
**FVC**	0.09		
DV_QB_ (L)		–0.27	0.01*
Gender		0.16	0.14
**FEV_1_**	0.09		
DV_QB_ (L)		–0.34	0.005*
DV_QB_ (R)		0.25	0.036*
**Bowel function**			
**BFI**	0.26		
Age		0.26	0.02*
DV_QB_ (L)		–0.26	0.02*
DE_Sniff_ (L)		–0.46	0.001*
**PAC-QoL**	0.06		
DE_DB_ (L)		0.19	0.09
Age		0.27	0.02*
**Urological function**			
**ICIQ-OAB**	0.26		
DV_sniff_ (L)		0.41	< 0.001*
Age		0.34	0.001*
**IPSS**	0.32		
DTEE		–0.34	0.02*
Age		0.45	0.003*

*p < 0.05 presented in bold. COP, center of pressure; L, left; R, right; DV_Sniff_, diaphragm velocity during sniff test; PC, principal component; DE_Sniff_, diaphragmatic excursion during sniff test; FVC, forced vital capacity; DV_QB_, diaphragm contraction velocity during quiet breathing; FEV_1_, forced expiratory volume during the first second; DE_DB_, diaphragmatic excursion during deep breathing; BFI, Bowel Function Index; PAC-QoL, Patient Assessment of Constipation Quality of Life; ICIQ-OAB, International Consultation on Incontinence Questionnaire for Overactive Bladder; IPSS, International Prostate Symptom Score; DTEE, diaphragm thickness at end-expiratory.

## Discussion

In this study, we found that diaphragm function was influenced by gender in PD, which is consistent with other researches (Spiesshoefer et al., 2020; Tuinman et al., 2020). We also discovered that diaphragm function showed significant differences between H&Y stages, instead of motor subtypes in patients with PD. The result may be related to the fact that the diaphragm function in the earlier stage of PD showed more compensation ability than that in the later stage. The significant difference in diaphragm function in the progression of the disease may provide insight into the time window of rehabilitation. Besides, we identified correlations not only between the diaphragm and respiratory function but also between postural stability, voice, and intestinal and urological function in our cohort of patients. In the following, we would discuss how the diaphragm gets involved in the abovementioned physiological behaviors in sequence.

The result of the pulmonary function test showed that these patients should not be diagnosed with restrictive or obstructive ventilation dysfunction according to the Global Initiative for Chronic Obstructive Lung Disease (GOLD; Vogelmeier et al., 2017). Respiration is controlled by the active contraction of the diaphragm to expand the chest and lung and decrease the intrapulmonary pressure lower than atmospheric pressure. Obstructive and restrictive ventilation dysfunction may occur in patients with PD due to the reduced compliance of the rib cage or dystonia of the upper airway (Sabaté et al., 1996; Tsai et al., 2016). Considering the negative correlation between the right diaphragm contraction velocity and FVC or FEV_1_, we deemed that diaphragm may indirectly compensate for relatively poorer respiratory function. Therefore, advanced PD patients with respiratory dysfunction may benefit from diaphragm training.

Postural instability may be related to the increased flexor muscle tone of the trunk which interferes with the projection of the center of mass to the base of support (Schoneburg et al., 2013); a reduction of proprioception and an increase of fatigue of the musculoskeletal system may also involve (Schoneburg et al., 2013; Moretto et al., 2021). In our study, the regression model suggested that diaphragm contraction velocity and excursion were higher in patients with poorer levels of control. Contraction velocity is an indicator of respiratory muscle strength (Sarwal et al., 2013) while increased mobility may elevate intra-abdominal pressure to stabilize the trunk and stimulate mechanoreceptors in the diaphragm crura (Hodges and Gandevia, 2000; Kocjan et al., 2018). The diaphragm may alter to activate core muscles and compensate for flexed posture in patients with PD. Soilemezi et al. (2020) also reported that patients who failed a weaning trial exhibited a higher peak transdiaphragmatic pressure and peak contraction velocity than healthy volunteers. They hypothesized that such alterations may compensate for diaphragmatic perfusion and were beneficial for recovering optimal muscle length to initiate the following contraction. Similarly, Özkal et al. (2019) indicated that the diaphragm was thicker in the elderly compared with the younger adults to compensate for atrophic lower limbs and maintain balance. The relationship between diaphragm function and balance function was further confirmed in longitudinal clinical trials: diaphragmatic breathing training or dynamic neuromuscular stabilization was able to promote balance function (Stephens et al., 2017; Yoon et al., 2020).

The score of VHI-10 indicated general dysphonia in this cohort of patients (Ng et al., 2020). PD can cause numerous changes in a patient’s voice, including hypokinetic dysarthria and monotonic articulation (Ma et al., 2020). These deficits are due to incomplete closure of the glottis, rigidity of the laryngeal muscle, and the reduction of the expulsion of lung air volume per syllable (Hammer, 2013; Ma et al., 2020). Earlier studies proposed that the diaphragm could be regarded as separate functional units during phonation (Hammer, 2013; Traser et al., 2017, 2020). The posterior diaphragm was elevated to reduce lung volume during exhalation whereas the anterior diaphragm and rib cage remained in inhalation position. In this study, we found that VHI-10 decreased by 0.3 points for every unit increase in diaphragm excursion. The positive correlation between diaphragm and voice function implied that the patients with dysphonia tend to amplify diaphragmatic excursion to facilitate subglottal pressure or respiratory drive to enhance voice function.

The BFI score indicated that constipation was quite common among our cohort of patients (Madeo et al., 2015). Constipation appears to be caused by delayed colonic transit and paradoxical movements of the pelvic floor muscles in PD. In this study, we found that patients with less burden tend to reserve better diaphragm function. The impaired diaphragmatic function may be caused by the dysfunction of pelvic floor muscles in PD. The pelvic floor muscles operate with other synergistic muscles, such as the diaphragm and deep erectors, to maintain intra-abdominal pressure (Szczygiel et al., 2018; Hwang et al., 2021). It is reasonable to suggest that paradoxical movement of the pelvic floor muscles might impact the diaphragm. Correlations between the diaphragm and defecation have also been reported. For example, patients with diaphragm weakness often experience defecation difficulty; diaphragm pacing has also been shown to improve defecation in quadriplegic patients (Perry et al., 2010). PD Patients with constipation could benefit from respiratory rehabilitation by improving diaphragm excursion or contraction velocity, and an increase in contraction velocity may help to recruit diaphragmatic motor units and compensate for lower gastrointestinal peristalsis (Fogarty and Sieck, 2020).

The loss of dopaminergic neurons in the substantia nigra pars compacta is known to lead to the disinhibition of the micturition reflex in the pons, thus resulting in an overactive bladder in patients with PD (Jost, 2012; Pfeiffer, 2020). The role of the diaphragm in reducing urinary incontinence has been demonstrated in a randomized clinical trial which assigned patients with radical prostatectomy to diaphragm training or pelvic floor muscle training, and researchers observed comparable effects between groups (Zachovajeviene et al., 2019). The diaphragm, abdominal muscles, and pelvic floor muscles are known to be functionally linked by numerous myofascial connections (Mazur-Bialy et al., 2020). Previous studies have reported that the combination of pelvic floor muscle exercise and urge suppression and voiding schedules effectively reduced the frequency of micturition and the severity of OAB and also improved the QoL (McDonald et al., 2020). Other researchers have found that reducing abdominal fat and practicing diaphragmatic breathing could improve the severity of overactive bladders in young women (Hagovska et al., 2020). The underlying principle of these observations relates to the reduction of detrusor pressure, an increase in urethral pressure, and the inhibition of the urination reflex. We found that patients with higher OAB score tended to show better diaphragm function. We assumed that OAB in PD may stimulate the potential of the diaphragm to function as a whole to compensate for the impairment. With regard to IPSS, the diaphragm was thinner in patients with a severer symptom. Training of the diaphragm or pulmonary rehabilitation may be considered for patients with PD who suffered from urinary dysfunction.

Our data indicated that the diaphragm plays a role in postural stability and visceral function in PD. However, there are some limitations in our study. First, although diaphragm ultrasound has been used extensively in patients with respiratory disorders, this technique is not routinely applied for patients with PD. Consequently, the reliability and reproducibility of this technique need to be evaluated in patients with PD. Second, the diaphragm ultrasound was taken during the ON state; whether the diaphragm exerts different effects during the OFF state still needs to be clarified. Third, this study was exploratory; future longitudinal studies should be rigorously designed and recruit a larger number of participants to fully confirm the potential role of the diaphragm in PD.

## Conclusion

The diaphragm was influenced by gender and disease progression but not motor subtypes in patients with PD. Our investigations also demonstrated the correlations between the diaphragm and postural stability, vocal function, respiratory function, intestinal function, and urological function in PD.

## Data Availability Statement

The raw data supporting the conclusions of this article will be made available by the authors, without undue reservation.

## Ethics Statement

The studies involving human participants were reviewed and approved by the Ethics Committee of Beijing Rehabilitation Hospital of Capital Medical University (2018bkky022). The patients/participants provided their written informed consent to participate in this study.

## Author Contributions

B-YF, J-NX, H-YJ, and XY conceived and designed the study. Z-HY, LG, and C-XZ performed the experiments. R-DW, J-PF, YS, and C-XZ collected patients’ data. XY and B-YF performed the statistical analysis. XY wrote the first draft of the manuscript. B-YF, J-NX, and H-YJ reviewed and edited the manuscript. All authors contributed to the manuscript revision and read and approved the submitted version.

## Conflict of Interest

The authors declare that the research was conducted in the absence of any commercial or financial relationships that could be construed as a potential conflict of interest.

## Publisher’s Note

All claims expressed in this article are solely those of the authors and do not necessarily represent those of their affiliated organizations, or those of the publisher, the editors and the reviewers. Any product that may be evaluated in this article, or claim that may be made by its manufacturer, is not guaranteed or endorsed by the publisher.
