# Supplementary material for: The Role of the Diaphragm in Postural Stability and Visceral Function in Parkinson’s Disease
Source: Front Aging Neurosci. 2021 Dec 23;13:785020. doi: 10.3389/fnagi.2021.785020 (PMC8733584; doi:10.3389/fnagi.2021.785020)
Supplement: Supplementary file 1 [file Data_Sheet_1.docx]

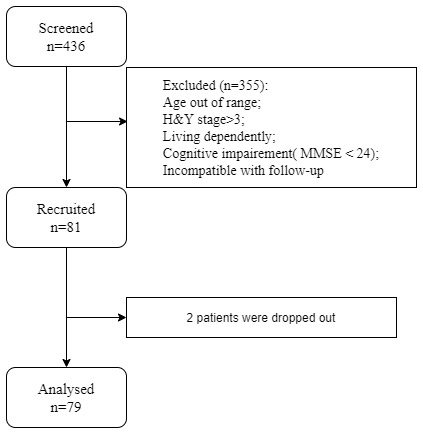


Supplementary Figure 1. Study Flow chart

Supplementary table1. Comparison of diaphragm function between different Parkinson’s disease motor subtypes

| **Variables** | **TD(n=27)** | **PIGD(n=45)** | **Indeterminate(n=8)** | ***P* value** |
| --- | --- | --- | --- | --- |
| DTEE (cm) | 0.21(0.08) | 0.22(0.09) | 0.22(0.13) | 0.996 |
| DETI (cm) | 0.34(0.12) | 0.36(0.15) | 0.32(0.28) | 0.924 |
| DTF | 66.25(44) | 61.54(48) | 53.59(25) | 0.818 |
| DE_QB_(R)(cm) | 1.41(0.52) | 1.18(0.67) | 1.15(0.49) | 0.889 |
| DE_Sniff_(R)(cm) | 4.75±1.65 | 4.17±1.39 | 3.99±1.16 | 0.238 |
| DE_DB_(R)(cm) | 4.8(1.98) | 4.8(1.58) | 4.71(2.55) | 0.642 |
| DV_QB_(R)(cm/s) | 1.08(0.38) | 1.00(0.29) | 1.14(0.35) | 0.642 |
| DV_Sniff_(R)(cm/s) | 4.18(2.27) | 3.67(1.8) | 3.47(1.25) | 0.285 |
| DE_QB_(L)(cm) | 1.21(0.5) | 1.24(0.63) | 1.20(0.6) | 0.989 |
| DE_Sniff_(L)(cm) | 4.34(1.6) | 3.83(2.08) | 3.29(1.04) | 0.359 |
| DE_DB_(L)(cm) | 4.35(1.45) | 4.23(1.73) | 3.45(2.4) | 0.367 |
| DV_QB_(L)(cm/s) | 1.09(0.33) | 1.05(0.39) | 0.93(0.51) | 0.499 |
| DV_Sniff_(L)(cm/s) | 3.88(1.57) | 3.66(1.54) | 3.49(2.27) | 0.299 |

Data are expressed as means ± standard deviation if normally distributed; otherwise, data are given as medians with interquartile range; A *p*<0.05, two-tailed was set as statistically significant. No significant difference was found using Kruskal Wallis test or ANOVA test.

Abbreviation: n, number; TD, tremor dominant; PIGD, postural instability/gait difficulty; DTEE，Diaphragm Thickness at End-Expiratory；DTEI; Diaphragm Thickness at End-Inspiration；DTF，Diaphragm Thickening Fraction；DE_QB_，diaphragmatic excursion during quiet breathing；DE_Sniff_，diaphragmatic excursion during Sniff test；DE_DB_，diaphragmatic excursion during deep breathing；DV_QB_，Diaphragm Velocity during Quiet Breathing；DV_Sniff_，Diaphragm Velocity during Sniff test

Supplementary table2. Right-to-left ratio during quiet breathing and deep breathing

| **Patient** | **Quite breathing** | **Deep breathing** | **Patient** | **Quite breathing** | **Deep breathing** |
| --- | --- | --- | --- | --- | --- |
| PD-01 | 1.1 | 1.1 | PD-41 | 1 | 1.1 |
| PD-02 | 1.9 | **2.2*** | PD-42 | 0.6 | 0.8 |
| PD-03 | 0.6 | 1.3 | PD-43 | 0.8 | 1.1 |
| PD-04 | 1.2 | 1.1 | PD-44 | 1.2 | 1.1 |
| PD-05 | 1.1 | **2*** | PD-45 | 1.3 | 1.3 |
| PD-06 | 1.2 | 1.2 | PD-46 | 0.9 | 1 |
| PD-07 | 1.2 | 0.8 | PD-47 | 1.3 | 1 |
| PD-08 | 0.8 | 0.8 | PD-48 | 1 | 0.9 |
| PD-09 | 0.5 | 1 | PD-49 | 1 | 0.8 |
| PD-10 | 1.7 | 1.1 | PD-50 | 0.9 | 1 |
| PD-11 | 2 | **1.8*** | PD-51 | 0.9 | 1.3 |
| PD-12 | 1.1 | 1.1 | PD-52 | 0.4 | 0.7 |
| PD-13 | 1.1 | 1 | PD-53 | 1 | 1.3 |
| PD-14 | 1.3 | 1.1 | PD-54 | 0.8 | 1 |
| PD-15 | 1 | 1.5 | PD-55 | 1.2 | 1.3 |
| PD-16 | 1.3 | 1.9 | PD-56 | 1.1 | 1.6 |
| PD-17 | 0.9 | 1.5 | PD-57 | 0.5 | 1.1 |
| PD-18 | 1.1 | 1.6 | PD-58 | 2 | **3.2*** |
| PD-19 | 1.7 | 1 | PD-59 | 1 | 1.1 |
| PD-20 | 0.9 | 1 | PD-60 | 0.8 | 1.2 |
| PD-21 | 0.9 | 0.9 | PD-61 | 0.9 | 1.4 |
| PD-22 | 1 | 1.1 | PD-62 | 0.6 | 1.8 |
| PD-23 | 1 | 1 | PD-63 | 1.1 | 1 |
| PD-24 | 0.8 | 0.9 | PD-64 | 1.1 | 1 |
| PD-25 | 1.1 | 1.1 | PD-65 | 1.5 | 1.7 |
| PD-26 | 1 | 0.9 | PD-66 | 1 | 1 |
| PD-27 | 1.1 | 1.2 | PD-67 | 1.1 | 0.9 |
| PD-28 | 1 | 0.8 | PD-68 | 1 | 1.4 |
| PD-29 | 0.8 | 0.7 | PD-69 | 1 | 1.2 |
| PD-30 | 1.1 | 1.5 | PD-70 | 1 | 0.8 |
| PD-31 | 1 | 1 | PD-71 | 0.7 | 1.1 |
| PD-32 | 1.9 | 1.5 | PD-72 | 1.6 | 1.2 |
| PD-33 | 1.1 | **1.6*** | PD-73 | 0.9 | 1 |
| PD-34 | 0.9 | 1.4 | PD-74 | 0.9 | 1.3 |
| PD-35 | 1.1 | 0.9 | PD-75 | 1 | 1.5 |
| PD-36 | 0.7 | 1 | PD-76 | 1.3 | 1.2 |
| PD-37 | 0.9 | 1.2 | PD-77 | 1 | 1.1 |
| PD-38 | 0.8 | 1.1 | PD-78 | 1.2 | 0.8 |
| PD-39 | 1 | 0.9 | PD-79 | 0.9 | 1.1 |
| PD-40 | 1 | 1.4 |  |  |  |

Normal range: 0.5-2.5 (quiet breathing); 0.5-1.6 (deep breathing), see reference (Sarwal et al., 2013);

Abnormal values are presented in bold; Abbreviation: R, right; L, light


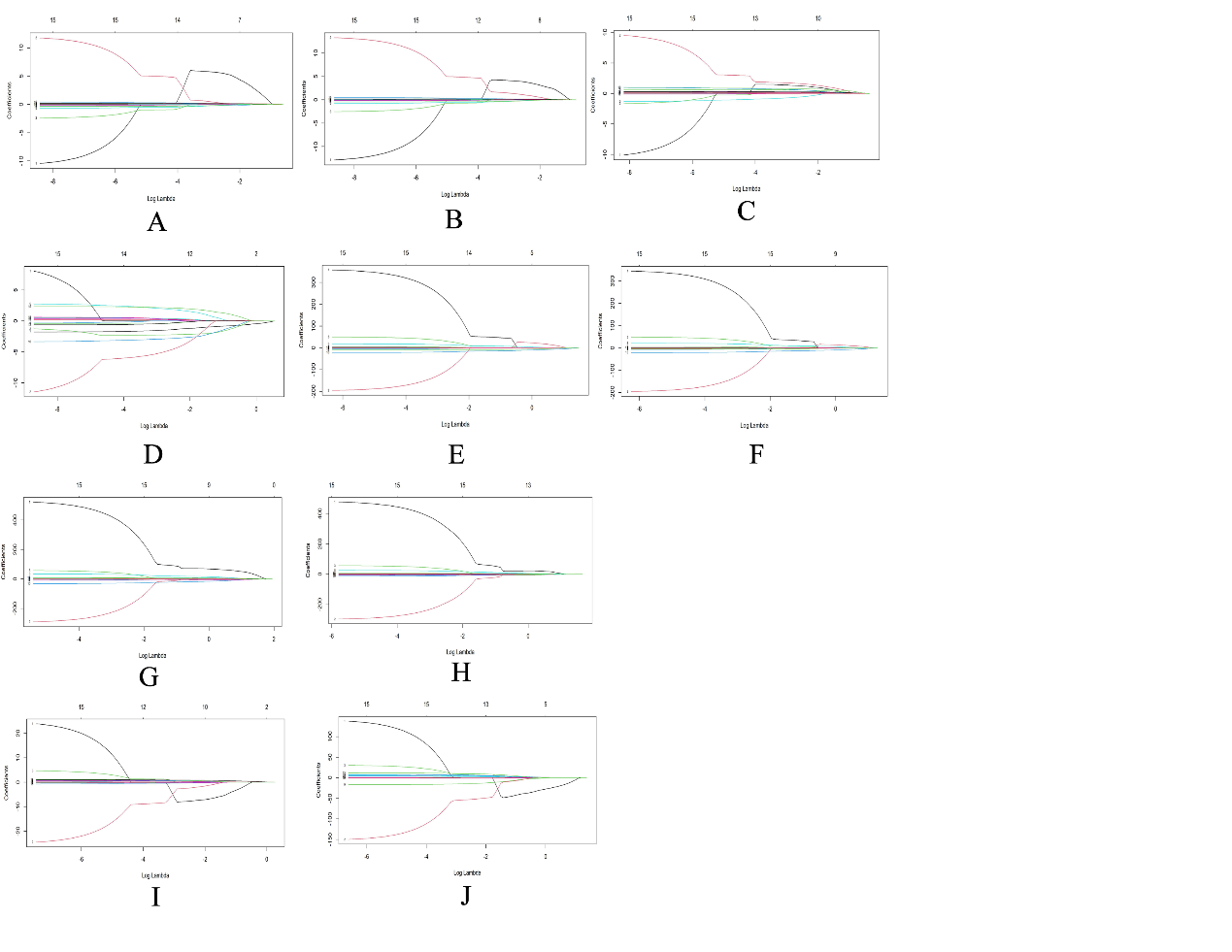


Supplementary Figure 2. LASSO regression model related to postural stability during eyes open (A), eyes close (B), tandem standing (C), voice function (D), FVC (E), FEV_1_ (F), bowel function index (G), Patient Assessment of Constipation Quality of Life (H), International Consultation on Incontinence Questionnaire for Overactive Bladder (I), International Prostate Symptom Score (J)

Supplementary table 3. Diaphragmatic variables selected by LASSO regression and principal component analysis

|  | **Coefficient** | **Standard Error** | ***P* value** |
| --- | --- | --- | --- |
| **Postural stability (COP)** |  |  |  |
| **Eyes open** |  |  |  |
| DV_Sniff_(R) | 0.31 | 0.11 | **0.01 *** |
| **Eyes close** |  |  |  |
| DV_Sniff_(R) | 0.24 | 0.10 | **0.01 *** |
| **Tandem standing** |  |  |  |
| PC 1 | 0.62 | 0.18 | **0.00 *** |
| **Voice Function** |  |  |  |
| DE_Sniff_(R) | -1.06 | 0.44 | **0.02 *** |
| **Respiratory Function** |  |  |  |
| **FVC** |  |  |  |
| DV_QB_(L) | -14.98 | 4.94 | **0.00 *** |
| Gender | -9.53 | 3.47 | **0.01 *** |
| **FEV_1_** |  |  |  |
| DV_QB_(R) | 11.31 | 5.47 | **0.04 *** |
| DV_QB_(L) | -13.66 | 4.93 | **0.01 *** |
| **Bowel Function** |  |  |  |
| **BFI** |  |  |  |
| DV_QB_(R) | 21.67 | 9.75 | **0.03 *** |
| DV_QB_(L) | -22.53 | 8.86 | **0.01 *** |
| DE_DB_(L) | 9.03 | 2.89 | **0.00 *** |
| DE_Sniff_(L) | -8.64 | 3.52 | **0.02 *** |
| DV_Sniff_(L) | 7.29 | 2.28 | **0.00 *** |
| Age | 0.83 | 0.39 | **0.04 *** |
| **PAC-QoL** |  |  |  |
| DE_DB_(L) | 6.70 | 2.29 | **0.00 *** |
| Age | 0.65 | 0.31 | **0.04 *** |
| **Urological Function** |  |  |  |
| **ICIQ-OAB** |  |  |  |
| DV_Sniff_(L) | 0.76 | 0.23 | **0.00 *** |
| Age | 0.17 | 0.04 | **0.00 *** |
| **IPSS** |  |  |  |
| DTEE | -39.44 | 17.88 | **0.03 *** |
| Age | 0.38 | 0.12 | **0.00 *** |

**P*<0.05 were presented with bold. Abbreviation: COP, center of pressure; L, left; R, right; DV_Sniff_, diaphragm velocity during sniff test; PC, principal component; DE_Sniff_, diaphragmatic excursion during sniff test; FVC, forced vital capacity; DV_QB_, Diaphragm Contraction Velocity during Quiet Breathing; FEV_1_, Forced Expiratory Volume during the first second; DE_DB_, diaphragmatic excursion during deep breathing; BFI, Bowel Function Index; PAC-QoL, Patient Assessment of Constipation Quality of Life; ICIQ-OAB, International Consultation on Incontinence Questionnaire for Overactive Bladder; IPSS, International Prostate Symptom Score; DTEE, Diaphragm Thickness at End-Expiratory

Supplementary table 4. Pearson correlation analysis between diaphragm and postural stability and Visceral Function

| **r/*p*** | **DV_Sniff_(R)** | **PC 1** | **DE_Sniff_(R)** | **DV_QB_(R)** | **DV_QB_(L)** | **DE_DB_(L)** | **DE_Sniff_(L)** | **DV_Sniff_(L)** | **DTEE** |
| --- | --- | --- | --- | --- | --- | --- | --- | --- | --- |
| **Postural Stability** |  |  |  |  |  |  |  |  |  |
| Eye open | **0.29/0.009;95%CI:0.08,0.48** | / | 0.15/0.19 | -0.09/0.45 | 0.05/0.68 | 0.18/0.12 | 0.12/0.28 | **0.25/0.03;95%CI: 0.03,0.45** | 0.22/0.06 |
| Eye closed | **0.28/0.01;95% CI: 0.06,0.47** | / | 0.18/0.12 | -.08/0.5 | 0.06/0.6 | 0.18/0.12 | 0.14/0.21 | **0.25/0.03; 95% CI:0.03,0.45** | **0.23/0.04; 95% CI:0.01,0.43** |
| Tandem standing | **0.31/0.005;95%CI: 0.1,0.5** | **0.4/0.0003;95% CI: 0.19-0.57** | **0.32/0.004; 95% CI:0.11, 0.5** | -0.005/0.96 | 0.21/0.06 | 0.2/0.08 | **0.25/0.03; 95% CI: 0.03, 0.45** | **0.28/0.01; 95% CI: 0.06, 0.47** | **0.24/0.04; 95% CI: 0.02, 0.43** |
| **Voice Function** |  |  |  |  |  |  |  |  |  |
| VHI-10 | -0.05/0.66 | **/** | **-0.3/0.007; 95% CI: -0.49, -0.09** | 0.05/0.69 | -0.14/0.23 | -0.11/0.34 | **-0.22/0.05; 95% CI: -0.42, -0.001** | -.11/0.33 | 0.002/0.98 |
| **Respiratory Function** |  |  |  |  |  |  |  |  |  |
| FVC | -0.02/0.86 | / | 0.08/0.51 | 0.06/0.63 | **-0.29/0.01;**  **95% CI: -0.48, -0.07** | 0.1/0.4 | 0.07/0.57 | -0.04/0.7 | 0.13/0.27 |
| FEV_1_ | -0.08/0.47 | / | 0.12/0.29 | 0.13/0.27 | **-0.25/0.03; 95% CI: -0.44, -0.03** | 0.03/0.77 | 0.08/0.48 | -0.12/0.29 | 0.14/0.23 |
| **Intestinal Function** |  |  |  |  |  |  |  |  |  |
| BFI | 0.1/0.37 | / | -0.21/0.07 | 0.1/0.4 | -0.12/0.31 | 0.11/0.32 | **-0.22/0.05; 95% CI: -0.42, -0.001** | 0.15/0.18 | 0.2/0.08 |
| PAC-QoL | 0.08/0.49 | **/** | **-0.26/0.02; 95% CI: -0.46, -0.04** | 0.07/0.53 | -0.06/0.57 | 0.11/0.32 | **-0.24/0.04; 95% CI: -0.44, -0.02** | 0.07/0.53 | 0.14/0.23 |
| **Urological Function** |  |  |  |  |  |  |  |  |  |
| ICIQ-OAB | 0.17/0.15 | / | -0.15/0.2 | -0.03/0.8 | 0.04/0.74 | -0.07/0.54 | -0.1/0.36 | **0.33/0.003; 95% CI: 0.12, 0.51** | -0.21/0.07 |
| I-PSS | 0.16/0.35 | / | -0.05/0.79 | -0.09/0.96 | -0.04/0.82 | -0.02/0.92 | -0.18/0.31 | 0.3/0.08 | **-0.39/0.02; 95% CI: -0.64, -0.07** |

Pearson product-moment correlation coefficient, *P*<0.05 was considered statistically significant which were shown in bold.

Abbreviation: CI: Confidence interval; VHI-10, Voice Handicap Index-10；FEV_1_, Forced Expiratory Volume during the first second; BFI, Bowel Function Index；PAC-QoL, Patient Assessment of Constipation Quality of Life；ICIQ-OAB, International Consultation on Incontinence Questionnaire for Overactive Bladder；I-PSS，International Prostate Symptom Score.

**References**

Sarwal, A., Walker, F.O., and Cartwright, M.S. (2013). Neuromuscular ultrasound for evaluation of the diaphragm. *Muscle Nerve* 47(3)**,** 319-329. doi: 10.1002/mus.23671.
